# Viruses infecting a warm water picoeukaryote shed light on spatial co-occurrence dynamics of marine viruses and their hosts

**DOI:** 10.1038/s41396-021-00989-9

**Published:** 2021-05-11

**Authors:** Charles Bachy, Charmaine C. M. Yung, David M. Needham, Maria Consuelo Gazitúa, Simon Roux, Alexander J. Limardo, Chang Jae Choi, Danielle M. Jorgens, Matthew B. Sullivan, Alexandra Z. Worden

**Affiliations:** 1 Monterey Bay Aquarium Research Institute, Moss Landing, CA USA; 2 Ocean EcoSystems Biology, GEOMAR Helmholtz Centre for Ocean Research, Kiel, Germany; 3 Department of Microbiology, Ohio State University, Columbus, OH USA; 4 Electron Microscope Lab, University of California, Berkeley, Berkeley, USA; 5 University of Kiel, Kiel, Germany; 6 Present Address: Department of Ocean Science, The Hong Kong University of Science and Technology, Hong Kong, People’s Republic of China; 7 Present Address: DOE Joint Genome Institute, Lawrence Berkeley National Laboratory, Berkeley, CA USA; 8 Present Address: Marine Science Institute, The University of texas at Austin, Port Aransas, TX USA

**Keywords:** Microbial biooceanography, Phylogenetics

## Abstract

The marine picoeukaryote *Bathycoccus prasinos* has been considered a cosmopolitan alga, although recent studies indicate two ecotypes exist, Clade BI (*B. prasinos*) and Clade BII. Viruses that infect *Bathycoccus* Clade BI are known (BpVs), but not that infect BII. We isolated three dsDNA prasinoviruses from the Sargasso Sea against Clade BII isolate RCC716. The BII-Vs do not infect BI, and two (BII-V2 and BII-V3) have larger genomes (~210 kb) than BI-Viruses and BII-V1. BII-Vs share ~90% of their proteins, and between 65% to 83% of their proteins with sequenced BpVs. Phylogenomic reconstructions and PolB analyses establish close-relatedness of BII-V2 and BII-V3, yet BII-V2 has 10-fold higher infectivity and induces greater mortality on host isolate RCC716. BII-V1 is more distant, has a shorter latent period, and infects both available BII isolates, RCC716 and RCC715, while BII-V2 and BII-V3 do not exhibit productive infection of the latter in our experiments. Global metagenome analyses show Clade BI and BII algal relative abundances correlate positively with their respective viruses. The distributions delineate BI/BpVs as occupying lower temperature mesotrophic and coastal systems, whereas BII/BII-Vs occupy warmer temperature, higher salinity ecosystems. Accordingly, with molecular diagnostic support, we name Clade BII *Bathycoccus calidus* sp. nov. and propose that molecular diversity within this new species likely connects to the differentiated host-virus dynamics observed in our time course experiments. Overall, the tightly linked biogeography of *Bathycoccus* host and virus clades observed herein supports species-level host specificity, with strain-level variations in infection parameters.

## Introduction

Viruses are thought to play a major role in the control of phytoplankton populations in marine ecosystems [1, 2]. Exploratory surveys, aiming at characterisation of the gene content of the viral fraction (viromes), have been skewed towards recovering phage diversity, including well-characterised cyanophages, at basin, ocean, and global scales [3–7]. The number of culture-dependent genomes available from viruses infecting photosynthetic marine eukaryotic microbes (protists) is one order of magnitude less than for marine phages [8, 9]. The description of marine viral genomic diversity is therefore challenging, in part because many protists are difficult to grow, precluding isolation of their viruses by classical methods [10]. The resulting paucity of reference genomic data from viruses with known protistan hosts restricts the use of metagenomic and metatranscriptomic data for studying viral biodiversity and host-virus distributions.

*Bathycoccus* is a widespread picoeukaryotic (1.2–2.5 µm in length) prasinophyte alga, which is non-motile and covered by scales [11, 12]. *Bathycoccus prasinos* has a genome of 15 Mb and is phylogenetically related to two other genera of the Class Mamiellophyceae, *Ostreococcus*, its closest relative, and *Micromonas*, which is larger and motile [13–16]. It is now accepted that *Micromonas* and *Ostreococcus* each have considerable genetic diversity and multiple distinct genetic clades/species [17–21]. Because all *Bathycoccus* 18 S rRNA gene sequences from cultures and environmental surveys are thus far identical, *Bathycoccus* was long considered one species (*B. prasinos*). However, molecular environmental studies indicate there are at least two ecotypes (Clades BI and BII) that rarely co-occur [12, 22–24]. The Clade BI ecotype corresponds to the genome sequenced *B. prasinos* RCC1105, and is reported mostly in cooler mesotrophic and coastal systems. The Clade BII ecotype is solely represented by two isolates (RCC715 and RCC716) from the same Indian Ocean water sample, and has been shown by qPCR to be abundant in warmer, saltier environments, including oligotrophic subtropical gyres [12]. Partial genome sequences are available for the Clade BII ecotype, coming from a targeted metagenome [25] as well as single-cell metagenomes and traditional metagenomes [24]. The dearth of physiology studies on *Bathycoccus* Clade BII in comparison to other prasinophytes [26–30], including *B. prasinos*, reflects the difficulty in growing this open-ocean clade in an axenic, stable and reproducible manner.

A decade ago, viruses were isolated against the genome sequenced *B. prasinos* RCC1105 (Clade BI). These have genome sizes of 199 (BpV1) and 187 (BpV2) kilobase (kb) [31, 32] and phylogenetic analyses of the DNA polymerase B gene (PolB), placed them in a basal position relative to *Micromonas* and *Ostreococcus* viruses [25, 30–32]. All belong to the *Phycodnaviridae* family of dsDNA viruses of which those known to infect members of the Mamiellophyceae are termed prasinoviruses (genus *Prasinovirus*). PolB environmental surveys of prasinoviruses indicate *Bathycoccus* viruses are abundant in the Indian Ocean [33, 34]. These putative *Bathycoccus* viruses appear more diverse than other prasinoviruses, such that those in culture only represent a sliver of *Bathycoccus* viral diversity. No viruses have been reported that infect *Bathycoccus* isolates from warm and high salinity waters (i.e., Clade BII), suggesting they may comprise some of the reported unknown diversity.

We sampled the western Sargasso Sea near the Bermuda Atlantic Time-series Study (BATS) site [35], a system that is often highly stratified and nutrient-limited, to target viruses of *Bathycoccus* Clade BII [12, 23]. We recovered three new viruses, all of which infect Clade BII RCC716 and one of which infected Clade BII RCC715. Our studies then established their morphology, genomic features and phylogenetic relationships with viruses infecting *B. prasinos* and other prasinoviruses. Assays of their infection dynamics alongside analysis of their relative distributions in metagenomes indicate that these closely related viruses have substantially different ecological impacts and host specificity.

## Materials and methods

### Culture of host algal strains and viruses

*Bathycoccus* RCC715 and RCC716 were purchased from the Roscoff Culture Collection (Roscoff, France) and grown in semi-continuous batch cultures at 21 °C in L1 medium [36]. For viral isolation, seawater was collected at 32°10′59″N, 64°36′32″W on 10 April 2015 from the sub-surface chlorophyll maximum (80 m depth) using a Niskin bottle. A 500 ml subsample was sent to the lab on ice packs and 300 ml were filtered through a sterile Nalgene Rapid‐Flow 0.45 μm PES membrane filter (Thermo Scientific, USA) five days after collection. Viruses were concentrated ten times using 100,000 MWCO PES Vivaspin 20 spin filters (VS2042, Sartorius, DE) and then isolated using serial dilution procedures with exponentially growing RCC716 cultures. After a first dilution series using the concentrated seawater, three lysed RCC716 cultures were selected and used as three separate inocula for two subsequent rounds of end-point dilution-to-extinction. This led to recovery of three purified viral strains (i.e., non-mixed viral stocks). For purified stock preparations, we then inoculated exponentially growing RCC716 cultures (50 ml) with the viral stocks (termed BII viruses) at 1% (500 µl). Once cultures were cleared (by lysis, after four days for BII-V1 and ten days for BII-V2 and -V3), the 50 ml were filtered through 0.45 µm PES membrane filters. Filtrates were concentrated with Vivaspin 20 spin filters as above. The purified, concentrated “master” stocks were stored at 4 °C in the dark.

For propagation and experiments, fresh *Bathycoccus* Clade BII viruses were generated by a primary infection of exponentially growing RCC716 host cells from each master stock. The infected RCC716 culture was allowed to lyse until it cleared. A 0.45-μm pore-size PES membrane Nalgene Rapid-Flow Sterile Disposable Filter Unit was used to remove large cellular debris. The viral-size fraction was then concentrated from the filtered lysate and washed with buffer using a 100-kDa MWCO PES membrane VivaSpin20 ultrafiltration unit. Viral concentrates were stored at 4 °C, protected from light, and used within 1–2 days.

### Flow cytometry

Flow cytometric analyses were performed using an Influx (BD Biosciences, USA). Green fluorescence (520/35 nm band-pass), red fluorescence (692/40 nm band-pass), side scatter and forward scatter were normalised with 0.75 μm YG fluorescent beads (Polysciences, USA). Samples were analysed for 2 min at 25 µl min^−1^. *Bathycoccus* were enumerated using side scatter and red fluorescence. Viral abundances were measured as previously described [37], by diluting 100 to 1000 fold with 0.02 μm-filtered 1X TE buffer, staining with 0.5X SYBR Green I with 15 min incubation in the dark at room temperature before analysis for 4 min at 25 µl min^−1^. Listmodes were analysed using Winlist (version 8.0, Verity Software House).

### Cross-infectivity tests

Multiple Mamiellophyceae (*Bathycoccus* spp. RCC715, RCC716, CCMP1898, RCC4222, *M. commoda* RCC299, *O. lucimarinus* CCMP2972, *O. mediterraneus* RCC789, *Ostreococcus* sp. RCC788, *O. tauri* OTH95) were grown and tested at concentrations of ~5 × 10^6^ cells ml^−1^ using fresh viral concentrate, with about 10^9^ viral particles ml^−1^. In all, 50 μL virus concentrate was added to 150 μL of exponentially growing cells in triplicate 96-well microplates and incubated at normal growth conditions for two weeks. Cell lysis was assessed every few days (day 1, 3, 5, 7, 10 and 14) qualitatively by visual inspection and quantitatively by measuring optical density on a Spectramax 340PC plate reader (Molecular Devices, USA) at 750 nm.

### Infection dynamics

RCC715 and RCC716 were grown at 21 °C under a 14 h/10 h light/dark cycle in L1 medium (as above for the culture of host algal strains and viruses) at 120 μE m^−2^ s^−1^ PAR. Both strains were maintained in light-acclimated, mid-exponential growth for ≥10 generations before experiment initiation. Two days before the experiment started, cultures of each host were divided into triplicates. Infections were carried out at 5:1 virus:host ratio, with starting concentrations of 5.5 × 10^6^ cells ml^−1^ (RCC715) and 4.1 × 10^6^ cells ml^−1^ (RCC716). Viruses were added to host cultures and mixed in a final volume of 30 ml using triplicate flasks for each virus (BII-V1, BII-V2, BII-V3), while negative controls received 0.02-μm-filtered TE buffer. FCM samples were collected immediately after the introduction of virus and every two hours for 30 h. T-tests were computed in R [38] to determine whether the cell numbers or normalised chlorophyll-derived fluorescence of the infected culture started to diverge from that of control.

### Most probable number assay for infectivity of *Bathycoccus* viruses

Most probable number (MPN) assays were set up the day before experiments to assess infectivity of fresh viral concentrates. In all, 50 μl of serially-diluted (10^−3^ to 10^−10^) virus sample was added to 150 μl of exponentially growing host cells in triplicate 96-well microplates and incubated as above for two weeks. As above for the cross-infectivity tests, cell lysis was assessed visually every few days (day 1, 3, 5, 7, 10 and 14) and by measuring optical density using a Spectramax 340PC plate reader. The MPN of infective viruses in each concentrate was estimated from the proportion of virus-positive wells using the MPN_ver4.xls Excel spreadsheet [39]. Percent infectivity was calculated by comparing MPN-estimated abundances of infective viruses to the abundance of viral particles determined by flow cytometry. Multiplicity of Infection (MOI) was calculated by comparing the MPN-estimated abundance of infective viruses to the host cell abundance (by flow cytometry).

### High pressure freezing with freeze substitution

For host electron microscopy cell preparation, exponentially growing RCC716 cells were concentrated by centrifugation, then cell pellets were either loaded fresh or pre-mixed with L1 medium with 20% bovine serum album as a cryo-protectant and then loaded into 25- or 50-µm-deep high pressure freezing planchettes [40]. Freezing was done using a Bal-Tec HPM-010 high-pressure freezer (Bal-Tec AG). High pressure frozen planchets stored in liquid nitrogen were transferred to cryovials containing 1.5 ml of 1% osmium tetroxide and 0.1% uranyl acetate in acetone at liquid nitrogen temperature (−195 °C) before undergoing freeze substitution using the method described in McDonald and Webb [41]. Briefly, the cryovials containing fixative and cells were placed into a metal block under liquid nitrogen; the cold block was put into an insulated container such that the vials were horizontally oriented, liquid nitrogen was poured from the container and shaken on an orbital shaker operating at 125 rpm. After 3 h, the metal block warmed to 0 °C and the samples were rinsed five times in pure acetone before resin infiltration, during which the planchettes were separated from the samples and removed. Cells were infiltrated with Epon resin in increasing increments of 30% over 2–4 h periods and then exchanged with 100% Epon resin three times before finally being gently centrifuged to the bottom of a Beem capsule prior to baking in an oven set to 60 °C for polymerisation.

### Electron microscopy

Fresh *Bathycoccus* Clade II virus lysates (~10^9^ viral particles ml^−1^) passed through a sterile Nalgene Rapid‐Flow 0.45 μm PES membrane filter were used for imaging. Virus lysates (10 µl) were deposited onto formvar-coated 200 mesh copper TEM grids (Ted Pella, Redding, CA, USA) and incubated for 15 min at room temperature. The remaining volume was removed and an additional 10 µl of lysate deposited and incubated for 15 min. Grids were washed with distilled water twice, and negatively stained with 10 µl of 2% uranyl acetate for 15 sec. Samples were imaged on a FEI Tecnai G2 Spirit TEM at an acceleration voltage of 80 kV. Viral capsid diameters were measured using ImageJ 1.50i software [42]. For host micrography, 90 nm sections were collected on formvar-coated grids using a Reichert UltracutE ultramicrotome (Leica Microsystems, Germany). Sections were post-stained using 2% uranyl acetate in water and Reynold’s lead citrate prior to being imaged in an FEI Tecnai 12 TEM (FEI, Hillsboro, OR) operated at 120 kV. Images were recorded using a Gatan Ultrascan 1000 CCD with Digital Micrograph software (Gatan Inc., Pleasanton, CA).

### DNA extraction

For initial PCR-based analysis of PolB, we extracted viral DNA using Wizard columns (Promega, USA). For genome sequencing, 5 ml of each of the three viral stocks was filtered onto 0.1 µm, 45 mm Supor filters (Pall Scientific, USA) and flash-frozen in liquid nitrogen. This material was extracted using a modified CTAB protocol [15, 43] designed to maximise the capture of unfragmented DNA molecules. DNA was quantified using a Qubit with the dsDNA HS Assay kit (Invitrogen, USA).

### PolB amplification and phylogenetic analysis

For preliminary taxonomic identification of the isolated viruses, the VpolAS4 and VAAS1 primer set that target a PolB gene fragment in prasinoviruses was used [33]. This PCR was performed using an initial melt of 2 min at 94 °C followed by 35 cycles with a melting step of 94 °C for 15 sec, annealing at 50 °C for 30 sec, extension at 72 °C for 90 s, and a final elongation of 72 °C for 10 min. Amplicons were cloned into pCRII-TOPO (Invitrogen, Carlsbad, CA) and 12 colonies were Sanger sequenced bidirectionally using plasmid primers M13F and M13R. Cloned sequences were added to a reference database compiling representative PolB gene sequences from published genomes or isolated viruses. Sequences were aligned with MAFFT [44] using default parameters and FastTree [45] was used to reconstruct a Maximum Likelihood (ML) tree.

Subsequently, full-length PolB sequences were identified in coding sequences predicted from the novel *Bathycoccus* viral genome sequences and added to our reference database used for phylogenetics. These were used as tBLASTn queries against NCBI nr/nt and recovered environmental sequences with e-value < 10^−15^ (and covering at least 25% of the gene) were retained and deduplicated. For identifying prasinovirus PolB from Tara Oceans metagenome assemblies [46], we downloaded assemblies from the European Nucleotide Archive. From assemblies with >300,000 contigs, we predicted proteins with Prodigal [47] and then selected likely PolB sequences by BLASTp [48] searches against a representative set of NCLDV PolB sequences, keeping those with e-value < 10^−25^. A secondary BLASTp search of the NCBI nr database (downloaded April 2018) was used to identify likely prasinoviruses. We excluded hits to uncultured taxa and *Tetrabaeana socialis* and *Chlamydomonas* (which apparently contain an integrated NCLDV in their genomes) [49, 50] and selected only the remaining protein sequences with best hits to *Bathycoccus*, *Micromonas*, or *Ostreococcus* viruses (e-value < 10^−5^). PolB sequences from an Arctic metagenome identified as being potentially novel prasinoviruses [51] were also added. PolB sequences from chloroviruses were used as an outgroup. In the final dataset, we discarded sequences shorter than 130 aa and re-aligned all sequences using MAFFT with default parameters. Misaligned and/or false-positive sequences and those with long branches were removed based on preliminary phylogenetic reconstructions with FastTree (default parameters) [45], resulting in a final set of 199 sequences that were re-aligned using the accurate model (-slowni) in MAFFT; positions with more than 25% gaps were discarded. The masked alignment comprising 325 amino acid positions was analysed using ML methods in RAxML [52] and the evolutionary model (LG + G + I) identified as appropriate using ModelTest-NG based on the corrected Akaike information criterion [53]. Node support was computed with 1000 bootstrap replicates.

### Genome sequencing, assembly and annotation

Libraries were prepared from CTAB DNA extracts using the NexteraXT DNA Library Preparation Kit (Illumina) according to the manufacturer’s instructions, and sequenced using the NextSeq platform. Assembly was performed on with the ~150 bp reads using SPAdes (v3.6.1), with the “single-cell” option activated and all other parameters set to their default value [54]. We considered that a viral genome was complete if it was assembled in one scaffold that could be circularised, i.e., included direct terminal repeats. However, this exercise is not meant to imply that the genome is circular, as PFGE indicates the BpVs [32] are linear, and the viruses in hand may be as well. We predicted genes with MetaGeneAnnotator [55]. All translated amino acid sequences were used in a BLASTp search [48] of the NCBI viral protein database (RefSeqVirus) for taxonomic affiliation (e-value<10^−3^ and bit score>50). Functional annotations were derived from the PFAM database of protein domains [56] using hmmsearch (e-value<10^−5^; [57]).

For comparative genomic analysis, predicted proteins from all the prasinoviruses and related chloroviruses were downloaded from NCBI (Supplementary information table S1). We defined orthogroups using Orthofinder default settings with the option -M msa which inferred gene trees using FastTree from multiple sequence alignments [58]. Hierarchical clustering of the viruses based on Euclidean distance was performed based on the presence/absence pattern of orthogroups using the pvclust R package [59] with otherwise default parameters (i.e., average linkage). To compare protein functional categories, we annotated each virus via the EggNOG pipeline [60] by searching the bacterial, archaeal, and eukaryotic databases independently and then selecting the annotation with the lowest e-value and a minimum seed_ortholog_score of 30. Hierarchical clustering of the viruses was based on the average linkage of the distribution of the functional categories using pvclust. The frequency of each category across the viral genomes determines how genes were clustered using the superheat R package [61] (i.e. complete linkage of Euclidean distances). The AAI-Matrix genome-based distance calculator programme [62] was used to estimate average amino acid identities between predicted coding sequences of *Bathycoccus* viruses with default parameters. Comparisons of *Bathycoccus* virus genome sequences were generated using progressive Mauve genome aligner version 2.3.1 [63] with default settings.

### Phylogenomics

For phylogenomic analyses, we extracted 22 core genes from the new genomes that span prasinoviruses and chloroviruses [64]. Predicted proteins from each core gene were individually aligned with MAFFT, and manually trimmed to discard long extremities and gap positions (those not found were considered as missing data). The individual protein alignments were concatenated to a single alignment comprising 7001 amino acid positions and analysed using ML methods under the LG + G + F model in RAxML as in [64, 65]. Node support was computed with 1000 bootstrap replicates. *Chlorella* viruses [66–69] were used as outgroup sequences to root the tree for display purposes only. Bayesian inference analysis was performed with MrBayes [70] implemented in Geneious v.8.0 [71], with two independent runs and 5,000,000 generations each. After checking convergence and eliminating the first 5000 trees, a consensus tree was constructed from sampling every 100 trees. The Bayesian tree confirmed the topology generated from ML reconstruction with branch support via Bayesian posterior probabilities of 1 (i.e., full support).

### Metagenomic analyses

To investigate distributions of the new and previously reported *Bathycoccus* viruses, we mapped reads from the 90 Tara Oceans Viromes [4]. To build the genomic references for recruitment of prasinovirus reads, we used nucleotide sequences of the 22 core genes (see above; taken from all available prasinovirus genomes), which each diverged by more than the 95% nt identity shared between *Bathycoccus* viruses. Using Bowtie2 [72], the reads were mapped competitively, filtered with a custom script to only retain alignment between the read and the reference genomes with a minimum cutoff of 95% identity, and the corresponding bam files manually checked for each virus, to ensure no read was recruited by two different viral genomes with identical similarity. Coverage values were calculated as reads mapped to the gene per kilobase of gene per million (RPKM) of metagenome reads. For the *Bathycoccus* Clade BII and *Bathycoccus prasinos* (Clade BI) hosts, we used a prior read recruitment analysis [24] for Tara Oceans samples for which the mapped metagenomic datasets had a corresponding virome sequenced, 54 samples in total (Supplementary information table S2). We focused on relative proportions between the various *Bathycoccus* hosts and *Bathycoccus* viruses independently, as the datasets were not obtained from the same size-fraction nor had the same sequencing depth. Spearman correlation analyses were performed in R [38] using both RPKM and proportion data and, we applied the Bonferroni correction to account for the small number of pairwise statistical tests. Non-parametric Mann–Whitney tests were computed in R to compare temperatures between two independent sets of samples. Canonical correspondence analysis in vegan [73] was used to visualise the spatial distribution of *Bathycoccus* hosts and viruses, and to identify the environmental factors that were most closely associated with their distributions. Matrices of the relative abundance of host and viruses at different locations were related to a constrained set of environmental variables (determined using the ‘step’ function in vegan [73] and Akaike information criterion, 999 permutations per step).

## Results and discussion

### Isolation and characterisation of viruses infecting the picoeukaryote *Bathycoccus* Clade BII

**Fig. 1:**
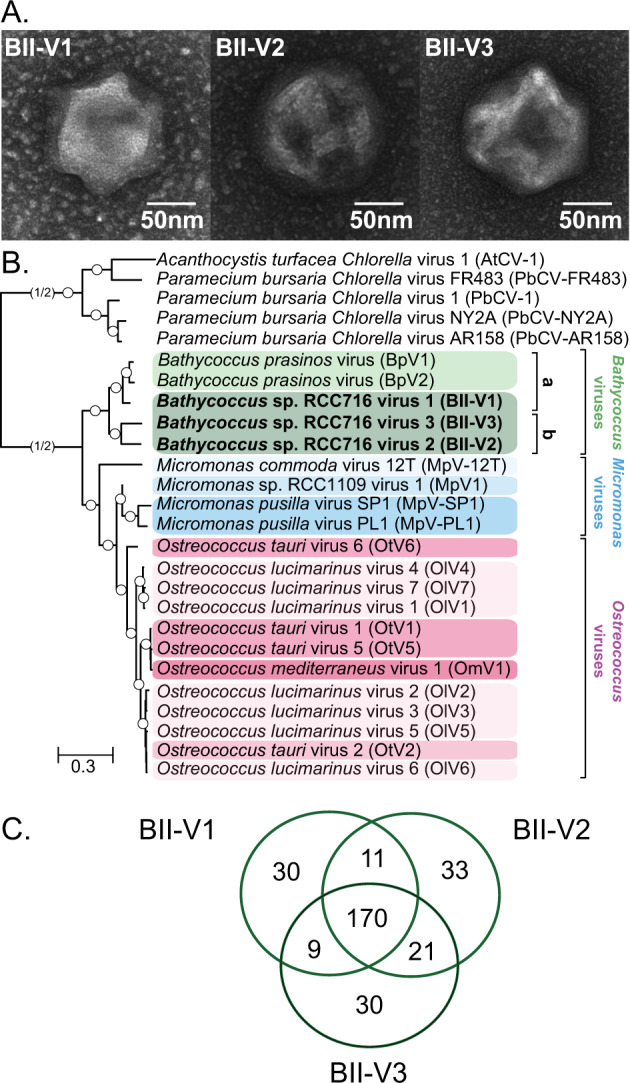
Morphology and evolutionary relationships of newly discovered *Bathycoccus* viruses. **A** Transmission electron micrographs of BII-V1, BII-V2 and BII-V3 (scale bar, 50 nm). The capsid diameters (*n* = 6 virions) measured 138 ± 2 nm (BII-V1), 150 ± 5 nm (BII-V2) and 152 ± 11 nm (BII-V3). **B** Maximum Likelihood (ML) phylogenetic reconstruction of green algal viruses inferred from a concatenated alignment of 22 core proteins shared among the viruses (7,001 positions) under the LG + G + F model. Node support was calculated from 1000 bootstrap (BS) replicates, with all branches acquired support values of 100% (white dots). Viruses infecting *Chlorella* were used as an outgroup and the branch connecting the prasinoviruses to the outgroup was truncated for display purpose. The new *Bathycoccus* viruses isolated against *Bathycoccus* Clade II (sensu [12]) isolate RCC716 (named as species *Bathycoccus calidus* herein, see below) are in bold. Colours reflect different host species within each genus. Letters alongside vertical lines (a and b) correspond to *Bathycoccus* viral clades. **C** Venn diagram of the shared and unique protein-encoding genes in the genome sequences of the new *Bathycoccus* viruses.

*Bathycoccus* BII isolates RCC716 and RCC715 used in our experiments were originally cultured from a nutrient-limited region in the Indian Ocean. Clade BII as a whole has been reported extensively in warm oligotrophic ocean gyres based on metagenome analyses [22–24]. Peak abundances occurr when well-developed deep chlorophyll maxima are present, or throughout the photic zone during mixing periods at Station ALOHA of the Hawaii Ocean Time-series [12]. We targeted BATS for viral isolation in springtime because *Bathycoccus* has been observed at relatively high abundance during this period using qPCR [74]. Here, three viruses were isolated against RCC716 [12] using seawater flown from BATS/Bermuda to the laboratory, obviating bringing this finicky strain into the field for use as a viral host. We then purified the viruses by serial dilutions and sequenced the partial PolB gene to determine whether they were evolutionarily different from other cultured viruses. BLASTn and preliminary phylogenetic analysis using GenBank nr sequences indicated they were distinct from described viruses with deposited sequences, with best BLASTn hits to *Bathycoccus prasinos* viruses (62–74% nucleotide identity). Transmission electron microscopy (TEM) revealed that all three viruses have similar morphology to other characterised prasinoviruses [75], with icosahedral capsids diameter ranging between 120 and 140 nm (Fig. 1A).

### Genomic sequencing and multi-gene evolutionary analyses

Assembly of DNA sequences from the viral isolates after deep sequencing by Illumina rendered one complete dsDNA genome sequence (BII-V3), and two others may still be partial (Table 1). The BII-V2 genome, which was in one contig, was similar in size (~208 kb) to that of BII-V3 (~212 kb). The BII-V1 genome assembly was ~174 kb and comprised of four linear dsDNA scaffolds. The viral concentrate was deeply sequenced (>50x coverage) and minor fragmentation of the genome was partially related to repeats that were not resolved during assembly. The total number of putative open reading frames (ORFs) varied from 220 in BII-V1 to 235 in BII-V2 (Table 1). Gene synteny was globally well-conserved across the BII-Vs and the BpV1 and BpV2 viruses of *B. prasinos* (Fig. S1), with limited genomic rearrangements. Other genome characteristics such as the coding proportion (~90%) and G + C % (~36%) were similar to other described prasinoviruses infecting Mamiellophyceae [64, 75], for which the reported number of proteins range from 203 to 268 and G + C % from 37 to 45%. However, the full-length PolB gene from the genome assemblies differed for BII-V3 from the other two, in having a 329 amino acid intein (Supplementary information table S3). Likewise, inteins have been reported at the same PolB position in uncultivated prasinoviruses from the subtropical Pacific Ocean [76], where *Bathycoccus* BII is abundant [12].

**Table 1:** Genomic characteristics of the three *Bathycoccus* viruses (BII-Vs) isolated against Clade BII isolate RCC716.

	Genome size (bp)	ORFs (no.)	G + C (%)	Gene density (gene per kb)	Coding proportion (%)	Average ORF size (bp)
BII-V1	174,426	220	35.2	1.27	89.5	705
BII-V2	207,870	235	36.5	1.13	92.7	820
BII-V3	211,597	230	36.3	1.08	91.2	844

*ORF* open reading frame.

To reconstruct a robust phylogeny for the new viruses, we employed 22 proteins previously identified as being shared across all available green algal virus genomes, including both prasinoviruses and chloroviruses [65]. We found all 22 in the predicted coding sequences of BII-V1; however, DNA helicase (SNF2) was not found in BII-V2 or -V3, FAD-dependent thymidylate synthase (thy1) and the topoisomerase IV were not found in BII-V2, nor was the prolyl 4-hydroxylase in the BII-V3 genome. Additional searches with tBLASTn did not recover these genes or fragments of them, suggesting they have been lost. Phylogenomic reconstruction grouped the three BII-Vs with the two BpVs [32], in a fully supported clade that branched adjacent to a large group of viruses that infect various species of *Ostreococcus* and *Micromonas* (Fig. 1B). The clade of *Bathycoccus* viruses was segregated in two subclades with BII-V2 and BII-V3 clustering together adjacent to BII-V1 and BpVs (Fig. 1B). While better resolution of the position of BII-V1 awaits greater taxonomic sampling, our results demonstrated that the three new viruses branch adjacent or basally to BpVs.

### Variation in prasinovirus gene content and functions encoded

**Fig. 2:**
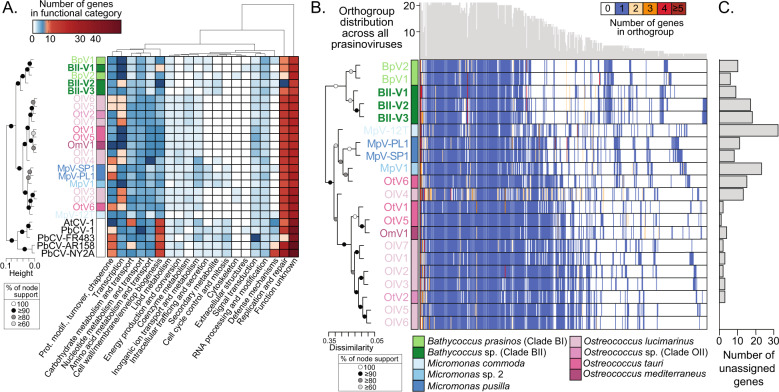
Distribution of functions and orthologous protein families across genome-sequenced prasinoviruses. **A** Functional category distributions across 21 genome-sequenced prasinoviruses and chloroviruses based on EggNOG categorisation. Viruses are clustered by similarity in their distribution of the functional categories on the *y*-axis and the frequency of each category across the viral genomes determines clustering along the *x*-axis ordering. Genes with homology to proteins in the EggNOG database but could not be assigned a function are in the “function unknown” category. **B** Orthogroups presence/absence patterns ordered along the *x*-axis by ranking according to the total number of genes in the orthogroup. For inclusion, the orthogroup was required to include protein sequences from at least two different viral genomes. Viruses are ordered along the vertical by their presence/absence pattern reconstructed by hierarchical clustering (topology on the left). Top histogram: frequency of each orthogroup in sequenced prasinoviruses. **C** Genes in each virus (number) not assigned to any orthogroup, with viruses in the same vertical order as **B**.

The three *Bathycoccus* Clade BII viruses had 72–77% of their proteins held in common, and ~30 unique proteins as well as a few proteins shared by just two of the three viruses (Fig. 1C). The 170 shared proteins had higher amino acid identities between BII-V2 and BII-V3 (73% aa identity) than to BII-V1 (69% and 68%, respectively). Generally, only 19–21% of *Bathycoccus* viral genes could be assigned a functional category, based on EggNOG classification. Similar functional category distributions were observed across both prasinoviruses and chloroviruses, including lipid metabolism, RNA processing and modification, and nucleotide metabolism and transport (Fig. 2A). Other functional categories were more variable, such as cell wall/membrane/envelope biogenesis genes prevalent in chloroviruses (potentially related to their enveloped nature), as well as genes involved in modification of the capsid with compounds such as with chitin and hyaluronan [77, 78] that are absent from prasinoviruses sequenced to date (Fig. 2A). Within prasinoviruses, most of the unique proteins in the *Bathycoccus* viruses lack defined functional categories. Among those with functional assignments, all five *Bathycoccus* viruses encoded a P2X receptor in the intracellular trafficking and secretion category, and both BII-V2 and -V3 encode two proteins putatively involved in degrading the aromatic compound 4-hydroxy-2-oxopentanoate to acetyl-CoA (secondary metabolite category), that otherwise are only encoded by one other prasinovirus, MpV1 [32]. Similar to the phylogenetic relationships, the functional category distributions of BII-V1 were closer to those of BpVs than to BII-Vs. The primary difference was in carbohydrate metabolism, where BII-V2 and -V3 each encodes ribulose-phosphate 3-epimerase (involved in the pentose phosphate pathway and carbon fixation; not found in any other available virus genomes, but encoded by *B. prasinos*) and TDP-glucose 4,6-dehydratase (involved in biosynthesis of rhamnose and encoded by most other chloroviruses and prasinoviruses [79]). Notably, the putative high-affinity phosphate transporter (PHO4, also termed HAPT) was only present in BII-V1 and BpV1, as well as OtV2 (isolated against the *Ostreococcus* Clade OII ecotype), and most sequenced viruses of *O. lucimarinus* (Supplementary information table S3). This gene is hypothesised to enhance phosphate uptake during infection under phosphorus‐limited host growth [25], as observed for the PstS phosphate transport system expressed by cyanophages [80], mitigating limitation of this key component of viral genomes. However, most isolated prasinovirus genomes come from waters that are not considered phosphate-limited, hence presence of this gene may connect to poising the host for responding to sudden availability of other nutrients, such as nitrogen, which is often limiting in the ecosystems from which these viruses were isolated. Studies of virus-cell responses under various limiting nutrients are required to understand the retention of this host-derived HGT.

Hierarchical clustering of orthologous proteins revealed patterns across prasinoviruses that generally corresponded with phylogenetic relationships. The BII- and Bp-viruses shared 130 orthologous proteins and hierarchical clustering (Fig. 2B) followed the clade structure of the phylogenomic reconstruction (Fig. 1B) with the exception of BII-V1 that grouped with BII-Vs, as well as OtV6, which grouped with *Micromonas* viruses. These orthologous proteins had on average 72% amino acid identity between BII-V2 and BII-V3, and 88% between the two *B. prasinos* viruses, but between 65 to 67% when comparing members of these two groups (Table 2). BII-V1 orthologs also had 67% and 66% amino acid identity to BII-V2 and BII-V3, respectively, while they had 83% and 80% identity to BpV1 and BpV2, respectively. Collectively, these results indicate that BII-V2 and -V3 diverged from BpVs prior to the divergence of BII-V1.

**Table 2:** Average percent amino acid identity of the orthologous proteins between the five *Bathycoccus* viruses.

Virus	Host	% identity to:				
		BpV1	BpV2	BII-V1	BII-V2	BII-V3
BpV1	*B. prasinos*	100	88.31	83.10	66.72	64.56
BpV2	*B. prasinos*	–	100	80.42	65.83	65.03
BII-V1	*Bathycoccus* RCC716	–	–	100	67.46	65.68
BII-V2	*Bathycoccus* RCC716	–	–	–	100	72.03
BII-V3	*Bathycoccus* RCC716	–	–	–	–	100

Of the 130 orthologous *Bathycoccus* virus proteins, 37% were assigned putative functions revealing core components of this viral group (Supplementary information table S3). These included genes involved in DNA replication and transcription, including PolB (type II), a DNA topoisomerase, a transcription factor S-II, mRNA capping enzymes, ribonucleases, a ribonucleotide reductase, and a dUTPase. Several others are necessary for viral particle synthesis, such as genes encoding structural elements for assembling the virion, including capsid proteins (5–6 copies per genome), as well as transcriptional regulators connected to the replication cycle. The BII viruses showed a number of differences among orthologous protein families. In addition to each having “unique” protein sets, there was a set of BII-V specific orthogroups, as well as some shared with BpVs, and/or other prasinoviruses (Fig. 1C and Supplementary information table S3). First, six predicted proteins showed orthologs across the three BII-Vs, but were not present in other prasinoviruses sequenced to date. Only one of these six was assigned putative function, belonging to the XRE family of transcriptional regulators. Additionally, all BII viruses harboured a tandem duplication of the FstH gene, while other sequenced prasinoviruses (including the two Clade BI viruses) have one copy (Supplementary information table S3). This ATP-dependent metalloprotease has been shown to be involved in photosystem II repair in cyanobacteria [81], and is present in genomes of photosynthetic eukaryotes, including all Mamiellophyceae [15, 16]. In *Arabidopsis* and *Chlamydomonas* it has been shown to be involved in protein quality control in the thylakoid membranes [82]. A gene of unknown function was also duplicated in the BII-virus genomes, that is a single copy in BpVs and absent from other sequenced prasinoviruses. Genes putatively encoding a glucose-1-phosphate adenylyltransferase, a glycosyltransferase and a thiamine pyrophosphate-requiring enzyme involved in amino acid biosynthesis were sporadically found in BII-viruses.

Considering the two *Bathycoccus* virus subclades (Fig. 1B), there is one predicted protein of unknown function exclusive to BpV1, BpV2 and BII-V1 and six predicted proteins shared only by BII-V2 and BII-V3. Among the latter, one belonged to the Ribulose-5-Phosphate-3-Epimerase (RPE) family, which catalyses the interconversion of D-ribulose 5-phosphate (Ru5P) into d-xylulose 5-phosphate, as part of the Calvin cycle (although no transit peptide was detected using TargetP) and in the oxidative pentose phosphate pathway. The ortholog analyses further showed that among prasinoviruses, 9, 17 and 18 genes were unique to BII-V1, BII-V2 and BII-V3, respectively (Fig. 2B). Apart from one nucleotidyltransferase and one glycosyltransferase (group 1) in BII-V1, none of these unique genes had known functions.

To study the evolutionary aspects of the shared prasinovirus proteins, we constructed and examined 130 phylogenies of orthogroups shared between *Bathycoccus* viruses. Nine showed a topology where all three BII-Vs grouped together with full support (100% bootstrap support), separate from the BpV orthologs, and in contrast to the multi-gene phylogeny where BII-V1 grouped with BpVs (Fig. 1B). The average amino acid similarities within each of these nine protein ortholog groups ranged from 85 to 88% between BII-Vs proteins, while they were 77 to 81% between BII-Vs and BpVs, different from overall amino acid similarity averages (Table 2). Interestingly, proteins from three of these nine ortholog groups, all lacking known functions, were adjacent to each other in the genome, or separated by only one gene. This synteny and co-location likely reflects the acquisition of these genes before co-infecting viruses diverged via viral recombination [83].

### Infection dynamics of *Bathycoccus* viruses

General host specificity of BII-viruses was assessed using two *B. prasinos* isolates (CCMP1898 and RCC4222, Clade BI), the two available Clade BII isolates (RCC715 and RCC716), four *Ostreococcus* species and one *Micromonas* species (Table 3). None were able to infect the *B. prasinos*, *Ostreococcus* or *Micromonas* isolates tested, suggesting BII-V specificity for *Bathycoccus* Clade BII. Similar host specificity has been observed in *O. lucimarinus* viruses, none of which infect *O. tauri* [64], and other viruses of eukaryotic and prokaryotic algae [84, 85]. Some other prasinoviruses appear to have broader host ranges [85–87], or their host species are less divergent than the two known *Bathycoccus* clades. For example, generalist viruses isolated against *Micromonas commoda* can infect *M. bravo* [85]. Further investigations are necessary to determine the extent to which the six shared proteins in BII-Vs (absent from BpVs), are responsible for the differences in host and virus specificity of interactions, versus variations in the shared *Bathycoccus* virus proteins (65–83% similarity). Importantly, host specificity tests for the new viruses described herein were limited by weak sampling of *Bathycoccus* diversity (in culture; all that we could acquire were tested).

**Table 3:** Results of cross infectivity tests of BII-V1, BII-V2 and BII-V3 against isolates representing various picoprasinophyte species within the Class Mamiellophyceae.

Strain #	Prasinophyte species	Strain isolation sea/ocean	Axenic	BII-V1	BII-V2	BII-V3
RCC715	*Bathycoccus* Clade BII	Indian	No	+	*	*
RCC716	*Bathycoccus* Clade BII	Indian	No	+	+	+
CCMP1898	*Bathycoccus prasinos* (BI)	Mediterranean	No	−	−	−
RCC4222	*Bathycoccus prasinos* (BI)	Mediterranean	No	−	−	−
RCC299	*Micromonas commoda*	Equatorial Pacific	Yes	−	−	−
CCMP2972	*Ostreococcus lucimarinus* (OI)	Eastern N. Pacific	Yes	−	−	−
RCC789	*Ostreococcus mediterraneus*	Mediterranean	No	−	−	−
RCC788	*Ostreococcus* Clade OII	Mediterranean	No	−	−	−
OTH95	*Ostreococcus tauri*	Med. Lagoon	Yes	−	−	−

Symbols: negative sign (−), no lysis; positive sign (+), lysis of the host tested and no regrowth after 14 days; asterisk (*), initial lysis but host cultures ultimately grew back on day 7.

Although specific for the BII clade, the three BII-Vs exhibited variations in infectivity of the two cultured BII strains available, despite their isolation from the same sample and having identical ITS1 and ITS2 sequences. BII-V1 lysed and cleared RCC715 and RCC716 cultures after four days (Table 3). The same was true for BII-V2 and BII-V3, when incubated with RCC716. Different from results for BII-V1, we found that while BII-V2 and -V3 initially lysed RCC715 cultures, resistant populations became evident at day 7 of infectivity tests, and measureable lysis of RCC715 could not be achieved thereafter. These results underscored the need to further examine host-virus interactions for the three new viruses.

**Fig. 3:**
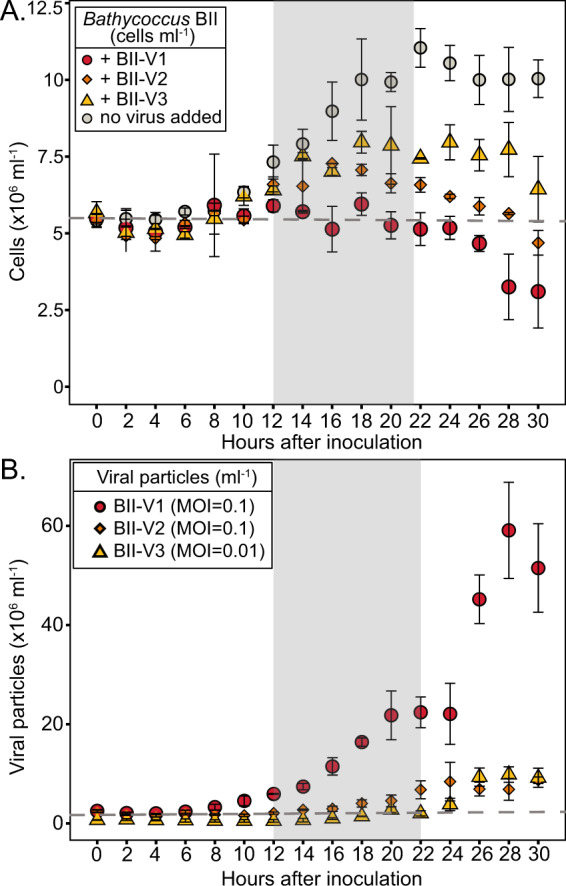
Temporal studies reveal differential infection dynamics. Flow cytometric enumeration of host cell abundance and viral particle abundance over experimental time courses. **A** Non-infected *Bathycoccus* Clade BII RCC716 control cultures (grey circles) as well as RCC716 cells in cultures inoculated with BII-V1 (red circles), BII-V2 (orange diamonds) and BII-V3 (yellow triangles). **B** symbols are as in **A** but represent the abundance of viral particles rather than hosts. Points and error bars represent mean values and standard deviation for biological triplicates. Horizontal grey dashed lines represent the cell or viral concentration at *T*_0_. Non-shaded and shaded areas represent light and dark periods, respectively. These experiments indicate BII-Vs are lytic viruses, although note that an intermediate state of chronic infection, without host lysis or integration into the host genome, has been described for *O. tauri* viruses [89].

Infection dynamics over time course experiments further illuminated differences in BII-V impacts on hosts. In these experiments, growth rates of the uninfected (control) RCC715 and RCC716 cultures were 0.45 ± 0.04 day^−1^ and 0.49 ± 0.06 per day, respectively, similar to rates during the pre-experiment acclimation period (T-test, *p* > 0.05). Host and virus dynamics were similar for RCC715 and RCC716 infected with BII-V1 (Fig. S2 and Fig. 3), with cell numbers starting to diverge from control abundances 10 h after inoculation (*T*-test, *p* < 0.05). Normalised chlorophyll-derived fluorescence of BII-V1 infected RCC715 and RCC716 was lower than in controls after two hours (Fig. S2C and Fig. S3A, *T*-test, *p* < 0.05), significantly in advance of host lysis and release of new viral particles which began 8-10 h after viral inoculation. Thus host physiology was markedly altered long before major cell lysis occurred.

Both BII-V2 and BII-V3 exhibited longer latent periods than BII-V1 in RCC716, such that host lysis and release of the new viral particles did not occur until 16–18 h after inoculation. Additionally, the estimated burst size of BII-V2 and BII-V3 (32 ± 3 and 140 ± 23 progeny virions per cell, respectively) was lower than that of BII-V1 (330 ± 267 progeny virions per cell, Supplementary information table S4). In all experiments, we strove to standardise the multiplicity of infection (MOI, ratio of infectious viruses to host cells), testing the MOI before and after each experiment. In these experiments, the measured MOI by MPN assays of BII-V3 (MOI = 0.01) deviated from initial characterisation, and as a result was lower than BII-V1 (MOI = 0.1) and BII-V2 (MOI = 0.1). This presumably made the contact rate of infectious BII-V3 and hosts lower, than for the other two viruses, however between BII-V1 and BII-V2 MOIs were consistent between experiments. Still, the viral-induced host mortalities by BII-V2 and BII-V3 at experiment end were 53 ± 7% and 35 ± 15%, lower than for BII-V1 (69 ± 13%; *T*-test, *p* < 0.01; Supplementary information table S5 and Fig. 3). Infectivity by prasinoviruses of *O. lucimarinus* also varies (reportedly from 14 to 32% infectivity during one less-controlled experiment) across closely related viruses that share higher average ortholog identity (90%) [30] than do the BII-Vs. Although it is tempting to compare with other phycodnaviruses, the host-virus models are often rapidly evolving systems and the methods are not consistent especially as many studies measure MOI once, at initial characterisation and not subsequently, and may, or may not, use a host strain grown in a defined state [88]. Our data show that a 10-fold variation occurs even between viruses that would typically be considered closely related. Thus, seemingly small genetic distances in common marker genes like PolB can reflect major differences in ecological impact of eukaryotic viruses.

Given that a resistant RCC715 population proliferated during BII-V2 and BII-V3 cross-infectivity tests, we expected infection dynamics of viruses inoculated into RCC715 cultures to differ from RCC716 experiments. Indeed, the MOI of BII-V2 (MOI = 0.004) and BII-V3 (MOI = 0.001) during RCC715 experiments were at least an order lower than in RCC716 infections. Although RCC715 lysis was observed under BII-V2 and BII-V3 infections, cell abundance did not always differ significantly from uninfected controls (Fig. S2A). Resistance responses have been reported for *B. prasinos* inoculated with BpV2, and *M. pusilla* against MpV1 in batch cultures [89], where lysis was observed after viral inoculation, but a fraction of cells always survived. Collectively, these results may reflect how arms-race dynamics [90] could lead to a range of infection phenotypes in closely related viruses like the BII-Vs.

### Integrating evolutionary and experimental results

Our multi-gene and PolB phylogenies suggested BII-V1 was evolutionarily more closely related to *B. prasinos* viruses than to the BII-V2 and BII-V3 viruses (Fig. 1B). Such incongruencies between host and viral phylogenies have been observed in other prasinoviruses at a taxonomic level finer than the genus [87, 91], but are not well understood. Such incongruencies are often interpreted as reflecting host-switching events. Indeed, the *Bathycoccus* clades can overlap in the field [12], thus there does not appear to be an ecological barrier to host switching for *Bathycoccus* viruses, even though those isolated here show a high species- and strain-specificity. Moreover, our phylogenetic analyses of genes shared by *Bathycoccus* viruses resolved nine genes (including three that were co-localised in the genomes) that formed supported clades that separated BII-V proteins from the BpV proteins. These proteins could enable BII-V1 to infect Clade BII strains like BII-V2 and BII-V3, while it was evolutionary closer to BpVs. However, BII-V1 did not infect *B. prasinos* and multiple other scenarios are possible. Moreover, unlike the multi-gene phylogeny, the distribution of orthogroups (Fig. 2B) indicated a close relationship between BII-Vs in terms of gene content, wherein BII-V1 clusters with BII-V2 and -V3, and not with BpVs, contributing more complexity to understanding the mechanistic basis for the differences observed in virulence and potential resistance mechanisms.

There are several factors arising from prior studies that likely contribute to the differential patterns in infectivity and virulence of the BII-Vs on Clade BII members. First, variations in host resistance levels have been proposed to connect to the small outlier chromosomes (SOC), possessed by all Mamiellophyceae sequenced thus far, potentially linking to a virus-immune state based on a study of *O. tauri* and its viruses [92]. The SOC has fragmented genes and greater variation than other genomic regions across species, that could contribute to differing viral immune responses across strains. Although this region cannot be assembled from metagenomic data, and hence is not known for Clade BII members, mapping of metagenomic reads to the *B. prasinos* genome demonstrates microdiversity within *B. prasinos* populations [23]. Thus, the genomes of RCC716 and RCC715 might well possess SOC divergence that underpins observed differences in host-virus dynamics.

An important consideration with respect to the dynamics we observed is that field studies of specific genes point to a greater diversity within the *Bathycoccus* genus than currently recognised, including uncultured strains within the Clade BII that are more diverged than RCC716 and RCC715. In this case, while we isolated viruses against *Bathycoccus* RCC716 (i.e., BII-V1), among the BII-Vs there could either be different strategies, or they could be optimised for different but closely related hosts. In tropical Atlantic waters, a study using targeted metagenomics and subsequent PCR-based sequencing of the spliceosomal gene, PRP8, identified molecular diversity within *Bathycoccus* Clade BII, with two co-existing variants [22]. Additionally, among 13 *Bathycoccus* environmental clones annotated as *Bathycoccus* Clade BII rRNA/ITS sequences [12], four from the tropical Pacific and Atlantic (KY382370, KY382373, KY382374, KY368637) present two single nucleotide polymorphisms (see taxonomic revision section). Together with the analyses herein, an interpretation of these results is that BII-V1 dynamics arise from it being a lineage of BII viruses, with different host-optimisation than BII-V2 and -V3. In this scenario, addition of other BII-V1-like viruses would lead to a BII-V1-clade branching separately from both the BII-V2 and -V3-like viruses and *B. prasinos* viruses in both phylogenomic and orthology presence/absence analyses.

### Distributions of host and virus based on marine metagenome surveys

**Fig. 4:**
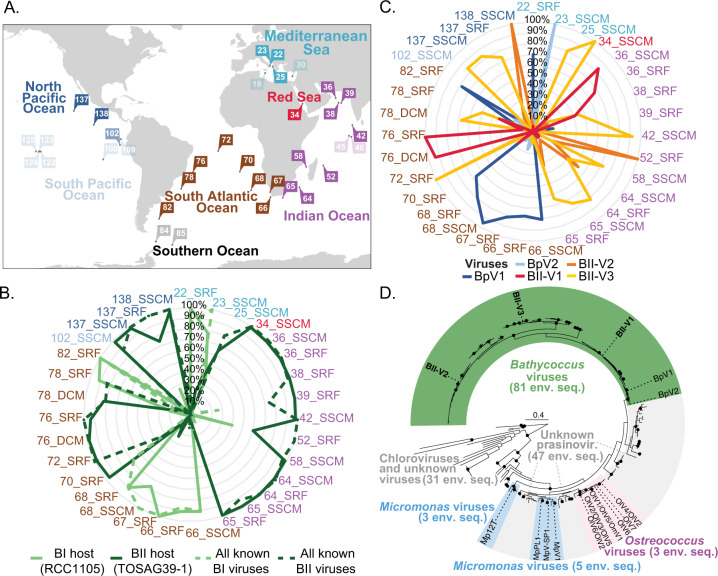
Global distribution and diversity of *Bathycoccus* species and viruses. **A** Map of Tara Oceans stations (modified from Sunagawa et al. [46]) from which metagenomic reads were recruited using the *B. prasinos* (BI), *Bathycoccus* RCC716 (BII), BII-V1, -V2 and -V3, as well as BpV1 and BpV2 genomes. Most stations contained reads from both the host and respective virus (solid colours) while in some only hosts reads were detected (muted colours). All stations in which any of the *Bathycoccus* viruses were detected also contained reads from the respective hosts. **B** Relative contribution of each *Bathycoccus* species (solid lines; BI, light green; BII dark green) based on [24] at Tara Oceans stations where we also detected viruses. Also shown are the relative contributions of viruses, computed as the sum of reads competitively recruited to genome-sequenced BpVs versus BII-Vs. **C** Relative contribution of reads competitively mapped against all *Bathycoccus* viruses. The colour code for stations is the same in all panels. SRF surface water, SSCM sub-surface chlorophyll maximum. **D** Phylogenetic reconstruction of prasinovirus PolB genes in GenBank nr and metagenomic data. ML reconstruction using the model LG + G + I based on a multiple sequence alignment of amino acids. Branches with BS values ≥90% (1000 replicates) are indicated (black dots). A total of 159 viral PolB sequences from Tara Oceans data were incorporated from assemblies with >300,000 contigs [46] along with 21 from viruses with known hosts and sequenced. Coloured blocks represent taxonomic classification of lineages based on supported clades (BS ≥ 90%) that include viruses with known hosts (only these are named on the tree). All labels and accessions are shown in Fig. S5.

The diversity and distributions of the specific marine eukaryotic hosts (prasinophytes and beyond) in connection to their viruses in the field have not yet been well characterised. Therefore, we next examined the distributions of the *Bathycoccus* viruses in nature, alongside the distributions of the two known *Bathycoccus* ecotypes, BI (*B. prasinos*) and BII (RCC716/715). Alongside a quantitative study demonstrating the two ecotypes occupy different marine water types, with some overlap [12], metagenome analyses show variations in the presence/absence of BI and BII in the Eastern North Pacific (ENP) [23] and Tara Oceans [24]. Further, sequences affiliated to previously available BpV sequences have been reported in cellular-fraction filtered seawater samples from the ENP and in Tara Oceans [22, 93]. We recruited reads from two Tara metagenome studies [4, 94] to *Bathycoccus* virus core genes (requiring 95% nucleotide identity) and compared their distributions to previously reported [24] relative abundances of *Bathycoccus* Clade BI and BII-affiliated reads in Tara data. One or both *Bathycoccus* Clades were detected in all 54 metagenomes analysed, while BI and BII viruses were detected in 31 out of 54 corresponding viromes (size fraction < 0.22 μm; Fig. 4A).

The distributions of the *Bathycoccus* Clades and their respective viruses showed strong co-occurrence patterns. Viruses infecting *B. prasinos* were concomitantly recovered with the *Bathycoccus* Clade BI host (Fig. 4B; Spearman’s correlation, *p*-value < 10^−5^, *r*^2^ = 0.61). Similarly, viruses infecting the *Bathycoccus* Clade BII were concomitantly recovered with the *Bathycoccus* Clade BII host (Spearman’s correlation, *p*-value < 10^−4^, *r*^2^ = 0.46). Out of the 31 samples where viruses and hosts were co-detected, the more abundant virus-type (BpVs or BII-Vs) corresponded with higher abundance of the known host clade in 29 of them. The other two stations were in frontal regions: Tara Station 70_SRF exhibited more BpV than BII-V (67% BpV of total *Bathycoccus* virus reads), while 96% of the *Bathycoccus* host reads were attributed to Clade BII (Fig. 4B); Tara Station 78-SRF, exhibited the opposite pattern with 83% BII-V comprising total *Bathycoccus* virus reads, while host reads predominantly mapped to *Bathycoccus* Clade BI (Fig. 4B). Stations 70, 72 and 76 are in the subtropical Atlantic basin and stations 68 and 78 sampled Agulhas rings from the South Atlantic Ocean (which behave as subpolar environments travelling across this subtropical region) [95]. Stations to the north (76, 72) have warmer waters and showed dominance of the BII host Clade, while the lower temperature adapted *B. prasinos* (BI) alone was detected in those to the south (82, 68). Mixing of *Bathycoccus* Clades has been reported in an eastern North Pacific transition zone [12], and supporting growth of multiple *Ostreococcus* Clades in Pacific transitional and frontal regions [96, 97]. Here, stations 70 and 78 appear to be similarly transitional, with the relative mapping abundances potentially reflecting differences of dispersion of free virions compared to host cells, or differences of viral decay rates or, from a technical perspective, statistical noise with the number of reads mapped in these two transitional samples that were lower compared to other viromes. To examine whether environmental factors would constrain *Bathycoccus* host and virus distribution, we performed a CCA using the relative abundance of *Bathycoccus* hosts and viruses alongside available environmental data (temperature, light, oxygen, salinity, phosphate and nitrite+nitrate). Temperature was identified as being significantly associated with the host and virus distribution (*p*-value < 0.01, Fig. S4), aligning with prior work indicating temperature, or a co-associated (but unmeasured) parameter, is one of the major drivers of the *Bathycoccus* ecotype distribution [12].

Ecologically the results further emphasise dynamics among different viruses and their epidemiology. Our analyses are based on mapping data to two previously available genomes for BI viruses and our three new BII viruses (all were isolated against cultured hosts), and therefore would not detect as yet uncharacterised/unsequenced viruses. Further, other viral genotypes could be present in metagenomic data, but not detected due to limitations connected to size-fractionation or depth of sequencing. Keeping these two caveats in mind, our results add a new dimension to environmental studies of eukaryotic algal viruses. Species-level specificity was previously observed in the field for viruses infecting *Ostreococcus* Clade OI, which flourishes in coastal and mesotrophic waters [97], but was not recovered in low latitude (warm) water samples [64] where *Ostreococcus* ecotype (Clade OII) usually thrives. Exploration of within-region dynamics showed that the haptophyte *Emiliania huxleyi* co-occurs with four to six different viral genotypes in the North Sea, but only two of these viruses dominated during and after blooms based on DGGE data from mesocosm studies [98]. In our study, the high intra-ecotype specificity observed, at least for the limited number of viruses isolated against members of the same *Bathycoccus* Clade, is notable. We found co-occurrences of different *Bathycoccus* viral types (two and sometimes three for BII viruses) infecting the same host Clade in 23 of the Tara Oceans samples analysed (Fig. 4C, Supplementary information table S6). Moreover, while BII-Vs often co-occurred in data from within individual sites (as did BpVs), generally only one viral type dominated (Fig. 4C). This is consistent with the Bank model [99], suggesting only a small fraction of a virus community is active and abundant at any given time while most populations are rare and dormant, forming a seed-bank that can ‘Kill-the-Winner’ when hosts reach critical abundance thresholds [100].

### Prasinovirus diversity in nature

We examined prasinovirus distributions in nature without a cultivation step by searching Tara Oceans metagenome assemblies [46] and other environmental data for related PolB sequences. One hundred and seventy three sequences longer than 130 aa affiliated to prasinoviruses using BLASTp and preliminary tree analyses. Phylogenetic analysis revealed that 51% of these belonged to *Bathycoccus* virus lineages, 6% were attributed to *Ostreococcus* and *Micromonas* viruses (Fig. 4D), and the rest could not be assigned to known prasinoviruses. Multiple *Bathycoccus* virus PolB sequences belonged to supported clades that contained the newly isolated viruses. BII-V1 and BII-V2 formed clades with nine and 14 environmental sequences (>98% bootstrap support), respectively (Fig. S5). BII-V3 formed a clade with eight environmental sequences, two of which contained almost identical PolB inteins to that in BII-V3. These two viral PolB sequences were recovered from subtropical/tropical North Pacific samples (stations 132 and 138). Members of the broader environmental BII-V3-like clade (21 sequences), lacked the intein. Inteins in the spliceosomal protein PRP8 of wild *Bathycoccus* BII [22] lack amino acid similarity with the viral inteins. Multiple other BII-V-like sequences grouped in clades related to those of the isolated BII-Vs. Overall the bulk of prasinovirus sequences recovered from existing metagenomics data were from BII-V related lineages, not from BpVs.

We observed that one PolB from cold Arctic waters (contig_35, Fig. S5) grouped with BII-V sequences. A prior study recovered *B. prasinos* metagenomic reads from the same sample and emphasised the possible importance of viruses in controlling *Bathycoccus* populations in the Amundsen Gulf [51]. Our findings of a BII-V affiliated sequence could reflect the presence of yet another undescribed *Bathycoccus* host lineage, adapted to cold waters, or a more generalist BII-V that can infect *B. prasinos*. However, high dispersion rates of BII viruses through transport from the North Pacific could also account for this result. Co-occurrence analyses of hosts and their specific viruses, with attention to currents and water mass transport, are needed to resolve this question.

Analysis of PolB diversity also identified viral clades that differed with respect to environmental parameters. Temperature at the time of collection differed between BII-V1 (24.4 ± 1.9 °C) and both BII-V2 and BII-V3 (19.2 ± 4.3^o^C and 19.12 ± 4.6^o^C, respectively; Mann-Whitney, *p* < 0.01) clades. Likewise, the BpV-related viruses were from significantly lower temperature waters (13.2 ± 8.6^o^C) than BII-V1, but not significantly different for BII-V2 and BII-V3. This mirrors CCA results from *Bathycoccus* host and virus distributions in metagenomics data and significant association with temperature (Fig. S4). Further, seven of the nine BII-V1 sequences came from the persistently warmest ocean in the world, the Indian Ocean, where the hosts we used for viral bait were also isolated. Additionally, two Indian Ocean studies noted diversity of potential *Bathycoccus* viruses, based on partial PolB gene sequences [33, 34]. Sequence comparisons with our data indicated these were BII-Vs (25 out of 28 *Bathycoccus* viral OTUs; data not shown) and only ~10% were BpVs, although the sequences were too short for inclusion in our phylogenetic reconstruction. We posit BII-V1 is a virus optimised for *Bathycoccus* Clade BII strains prevalent in warm waters (e.g., 28 °C) such as those at BATS in mid-summer. In contrast, BII-V2 and -V3 may specialise on another type of BII, or an as yet unidentified, but related *Bathycoccus* lineage at BATS in spring, when we collected our viral isolation sample. Here again, additional data collected with attention to temporal dynamics and ocean physics is needed to resolve drivers behind the observed patterns. The fact that viruses from a habitat rarely sampled in viral isolation efforts (i.e., open-ocean waters) revealed marked divergence, both from each other and from BpVs, emphasises the importance of such efforts for interpretation of sequence-based environmental surveys.

### Taxonomic revision of the genus *Bathycoccus*

**Fig. 5:**
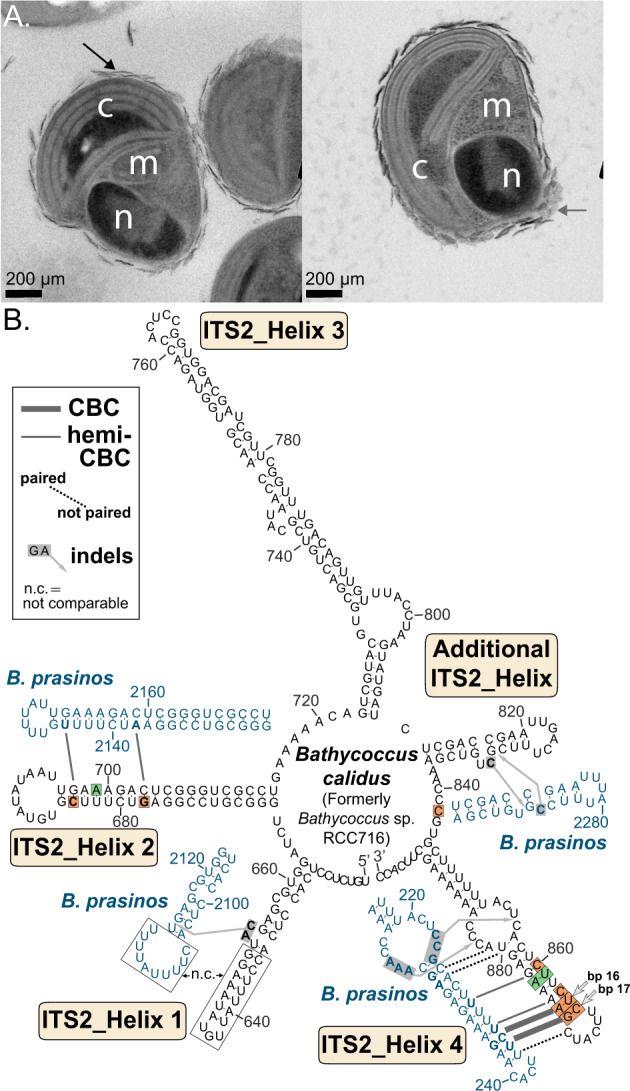
Cell morphologies and molecular signatures of novel species *Bathycoccus calidus*, formerly *Bathycoccus* RCC716, as compared to *Bathycoccus prasinos*. **A** Transmission electron micrographs of *B. calidus* isolate RCC716, reveal cell characteristics including, n nucleus, c chloroplast, m mitochondria, and arrows external scales. **B** The second internal transcribed spacer (ITS2) of *Bathycoccus calidus* (i.e., *Bathycoccus* Clade BII, KY563784) has ‘universal’ helices 1-4 and an additional helix (between helices 3 and 4), characteristic for the family Bathycoccaceae (i.e., *Bathycoccus* and *Ostreococcus* genera [103]). Helices of *B. calidus* were compared with homologous ITS2 helices from *B. prasinos* (Clade BI, JX625115) by highlighting compensatory base changes (CBCs), hemi-CBCs, base pairing/dissociation events (dotted lines), as well as hypothetical insertions/deletions (indels). A few regions, which were divergent and showed no intermediate evolutionary stages, were labelled as ‘not comparable’ (n.c.). Note that while the two Clade BII isolates have identical ITS sequences, among 13 *Bathycoccus* environmental rRNA/ITS sequences annotated as *Bathycoccus* Clade BII [12], four from tropical Pacific and Atlantic (KY382370, KY382373, KY382374, KY368637) present polymorphisms (green shading) at one nucleotide (helix 2, bp 16 reverse, G instead of A) as well as one deletion (helix 4, bp 13, bp not present). Tracing base pair evolution in the stem regions (i.e., helices) by CBCs and hemi-CBCs revealed several molecular signatures for *B. calidus* in helices 2 and 4, i.e., without homoplasies in the Bathycoccaceae [19], most of which evolved as hemi-CBCs. Signatures for *B. calidus* are indicated by orange shading. Base pairs 16 and 17 of helix 4 (corresponding to bp 18 and 19 in *B. prasinos*) were selected for the taxonomic diagnosis. A single-stranded nucleotide position in the conserved linker between the additional helix and helix 4 revealed another distinctive character of *B. calidus*.

The differences observed herein with respect to *Bathycoccus* virus distributions and viral exclusivity mirror support for the hosts, *Bathycoccus* Clade BII and *Bathycoccus* Clade BI, as being different species. Further, the Clade BII Internal Transcribed Spacer (ITS) sequences delineate it from Clade BI ITS [12] at a level generally considered appropriate for species designation. As observed by Limardo and collaborators (2017), and herein using different imaging methodologies (Fig. 5A), there is no apparent morphological differences between representatives from both clades. We, therefore, name strain RCC716 *Bathycoccus calidus* based on molecular diagnoses and the protocols of the International Code of Nomenclature for Algae, Fungi and Plants. The species name refers to RCC716 being isolated from warm (28^o^C) ocean waters, akin to distributions observed by qPCR [12], and metagenomics/transcriptomic read distributions [23, 24, 101]. This naming will avoid confusion arising from distributions of Clade BII and Clade BI being merged as “*B. prasinos*” patterns. BI is represented by the species *B. prasinos* [11] and was described prior to the availability of molecular data or isolation of RCC716.

Revision of the genus *Bathycoccus*

***Bathycoccus*** Eikrem & Throndsen, 1990, emend. Bachy, Yung and Worden.

Genus characteristics as previously described [11]. Three nuclear pores clustered at a single basal location similar to those in *O. tauri* [102]. Type species *Bathycoccus prasinos* Eikrem & Throndsen.

Emendation of the species ***Bathycoccus prasinos*** Eikrem & Throndsen, 1990, emend. Bachy, Yung and Worden.

*Description* – characters of the genus. Images and sequences describe the type specimen (CCMP1898). The latter are available in GenBank under the accession number JX625115 (partial nuclear-encoded rRNA transcriptional unit).

*Emended diagnosis* – in the ITS2 of the nuclear-encoded rRNA transcriptional unit, universal helix 1 contains a 10 nt loop composed of 5′-CUUUUAUUUU-3′ from positions 2104-2113 of JX625115 (Fig. 5B). Differences from other Mamiellophyceae are also exhibited in the ITS1 [103].

*Holotype* – strain CCMP1898, also known as SCCAP K-0417, is the type specimen and is preserved in a metabolically inactive state at the NCMA (https://ncma.bigelow.org/). It was isolated by J. Throndsen from the surface waters at 40°45′00″N, 14°19′48″E in the Gulf of Naples, Italy (17 April 1986).

*Habitat and ecology* – temperate to high-latitude marine waters and coastal regions. Distributed in samples analysed herein ranging from 8 to 25 °C and salinities of 30–35 ppt. Niche-specific Tara samples 7.3 to 17.0 °C (exclusively Clade BI, no Clade BII). Not detected in oligotrophic marine regions.

***Bathycoccus calidus*** Bachy, Yung and Worden, sp. nov.

*Description* − characters of the genus [11]. Sequences describe the holotype (RCC716) and are available in GenBank under the accession KY563784 (partial nuclear-encoded rRNA transcriptional unit).

*Validating illustration* – Fig. 5A and [12].

*Molecular diagnosis* − in the second internal transcribed spacer (ITS2) of the nuclear-encoded rRNA transcriptional unit, nucleotide 5 of the linker between the additional helix specific to Bathycoccaceae [103] and helix 4 is C, and base pairs 16/17 of helix 4 are U-A/C-G (Fig. 5B).

*Holotype* − cells of *B. calidus* strain RCC716 are preserved in a permanently metabolically inactive state for electron microscopy (in resin) in the University and Jepson Herbaria (UC/JEPS) at the University of California Berkeley (access number UC 2084460). DNA is also preserved (at −80 °C) in the Worden lab. RCC716 was collected on 6 November 2003 by Fabrice Not at 70 m depth in the Indian Ocean at a station (14°28’48”S, 113°27’00”E) with a surface temperature of 28 °C and purified from other algae by Florence Le Gall on 4 August 2003 using lab-based flow cytometry sorting. The living strain is maintained at the Roscoff Culture Collection (roscoff-culture-collection.org).

*Habitat and ecology* – present in warm oligotrophic ocean gyres, peak abundance typically in well-developed deep-chlorophyll maxima or throughout the photic zone during mixing periods. Distributed in samples analysed herein having salinities ranging from 33 to 36 ppt and temperatures from 10 to 29 °C (at the time of collection). Niche-specific Tara samples 18.6–27.7 °C (exclusively BII, no BI). Maximum reported abundance in multi-depth time-series at Station ALOHA occurred at 35 ppt, 23 °C [12].

## Conclusions

Our studies illustrate the value of combining culturing, genome analyses and physiology research with metagenomic studies. While it is well known that viruses influence the ecology and evolution of their eukaryotic hosts [25, 65, 104], little is known about eukaryotic host-virus diversity and distributions in marine ecosystems. Indeed, no viruses had yet been found that infected *B. calidus*, the species and type-strain we established for Clade BII, and considerable putative, but heretofore uninterpretable, *Bathycoccus* virus diversity has been reported. Our efforts to isolate and characterise unexplored open-ocean prasinoviruses revealed genome sequences from novel prasinoviruses that allowed identification and placement of novel viral diversity. Analysis of these new virus genomes, alongside existing *Bathycoccus* genomes and targeted metagenomes, elucidated virus and host connections to environmental conditions and niche specialisation. The observed generally contrasting distributions of the *Bathycoccus* species and their viruses highlight the importance of including temporal and physical processes in sampling of diversity or agents of mortality. Moreover, the host specificity and varied virulence levels of the three BII-Vs, and their environmental partitioning, illustrate an unrecognised level of microdiversity important for modelling host-virus dynamics and biogeography across the global ocean.

## Supplementary information

Legend Supplementary Material

Figure S1

Figure S2

Figure S3

Figure S4

Figure S5

Tables S1-S4-S5

Table S2

Table S3

Table S6

## Data Availability

The annotated genomes were submitted to GenBank under the accession numbers MK522034-MK522037 (BII-V1), MK522038 (BII-V2) and MK522039 (BII-V3). Sequence alignments for phylogenetic and phylogenomic analyses are deposited in TreeBASE. Cells of *B. calidus* strain RCC716 are deposited in the University and Jepson Herbaria (UC/JEPS) at the University of California Berkeley (access number UC 2084460).
