# Supplementary material for: Viruses infecting a warm water picoeukaryote shed light on spatial co-occurrence dynamics of marine viruses and their hosts
Source: ISME J. 2021 May 11;15(11):3129–47. doi: 10.1038/s41396-021-00989-9 (PMC8528832; doi:10.1038/s41396-021-00989-9)
Supplement: Supplementary file 1 — Legend Supplementary Material [file 41396_2021_989_MOESM1_ESM.docx]

**Supplementary Figures**

**Figure S1.** Colinearity of the five *Bathycoccus* virus genomes including the three that were isolated and genome sequenced in this study. Boxes with identical colors represent local colinear blocks (LCB), indicating homologous DNA regions shared by two or more genomes without sequence rearrangements.

**Figure S2.** Temporal dynamics of RCC715 viral infections. (A) Host cell abundance over time. (B) Viral particle abundance over time. (C) Bead normalized red fluorescence over time (as a proxy for chlorophyll content). (D) Bead normalized Forward Angle Light Scatter over time (as a proxy for cellular size). Gray circles represent non-infected control cultures, red circles represent infections with BII-V1, orange diamonds represent infections with BII-V2, and yellow triangles represent infections with BII-V3. Points and error bars represent mean values and standard deviation for biological triplicates, respectively. Dotted lines represent the cell or viral concentration at T_0_. Shaded areas indicate dark periods. The experimental design herein, involving high encounter frequencies, may not induce such other strategies, including exit from the cell without inducing lysis, when encounter frequencies are low.

**Figure S3.** Physiology of RCC716 during viral infections. (A) Bead normalized red fluorescence over time (as a proxy for chlorophyll content). (B) Bead normalized Forward Angle Light Scatter over time (as a proxy for cellular size). Gray circles represent non-infected control cultures, red circles represent infections with BII-V1, orange diamonds represent infections with BII-V2, and yellow triangles represent infections with BII-V3. Points and error bars represent mean values and standard deviation for biological triplicates, respectively. Dotted lines represent the cell or viral concentration at T_0_. White background indicates day period while shaded areas indicate dark periods.

**Figure S4.** Canonical correspondence analysis (CCA) biplot. CCA relating *Bathycoccus* host and virus distribution to environmental variables for each Tara Oceans sample. The percent of variation in the host and virus distribution explained by each axis is indicated in parentheses after the axis label. Environmental variables used in the CCA are represented by vectors, labels used in the figure are indicated by parentheses: water temperature (temp), light intensity (light) salinity, oxygen, phosphate (PO_4_), nitrate and nitrite (NO_2_NO_3_). Each circle represents the *Bathycoccus* host and virus composition at a specific location. Environmental variable marked with asterisks (only Temperature) are statistically significant (P<0.01), as assessed by the marginal effects of terms.

**Figure S5.** Phylogenetic reconstruction of the prasinovirus DNA polymerase B (PolB) gene. Here the same tree and colored clades as in main Fig. 4B with colored labels and circles for location and colored circles for latitudes. On the right, a panel with temperatures for each sample in which Tara Oceans environmental PolB sequences were detected and also the contig sequences from the Arctic metagenome [51] with the location of this polar sampling site on the upper right. Prasinovirus viral gene sequences that contain an intein are marked with a pink star. Amino-acid identity between the (three) *Bathycoccus* viral inteins ranged from 98.5% to 99.1%. Note that sequences from several PCR-based studies could not be included because they are partial sequences.

**Supplementary Tables**

**Table S1.** List of viral genomes used in the present study and publications first reporting them.

**Table S2.** List of Tara Oceans samples used for the metagenomics analyses.

**Table S3.** Orthologous protein-coding genes in BII-V1, BII-V2 and BII-V3 and orthologs detected in other prasinoviruses. Note that the 329 amino acid intein in the PolB is between YGD and TDS amino-acid motifs that corresponds to residue position 623 in the BII-V1_NODE1_gene_130.

**Table S4.** Summary of infection experiment parameters.

**Table S5.** Viral-induced host mortality.

**Table S6.** Reads per kilo base per million mapped reads (RPKM) for virally encoded core genes used in the metagenomic read recruitment analysis**.**
