# Supplementary figures and images for: Viruses infecting a warm water picoeukaryote shed light on spatial co-occurrence dynamics of marine viruses and their hosts
Source: ISME J. 2021 May 11;15(11):3129–47. doi: 10.1038/s41396-021-00989-9 (PMC8528832; doi:10.1038/s41396-021-00989-9)

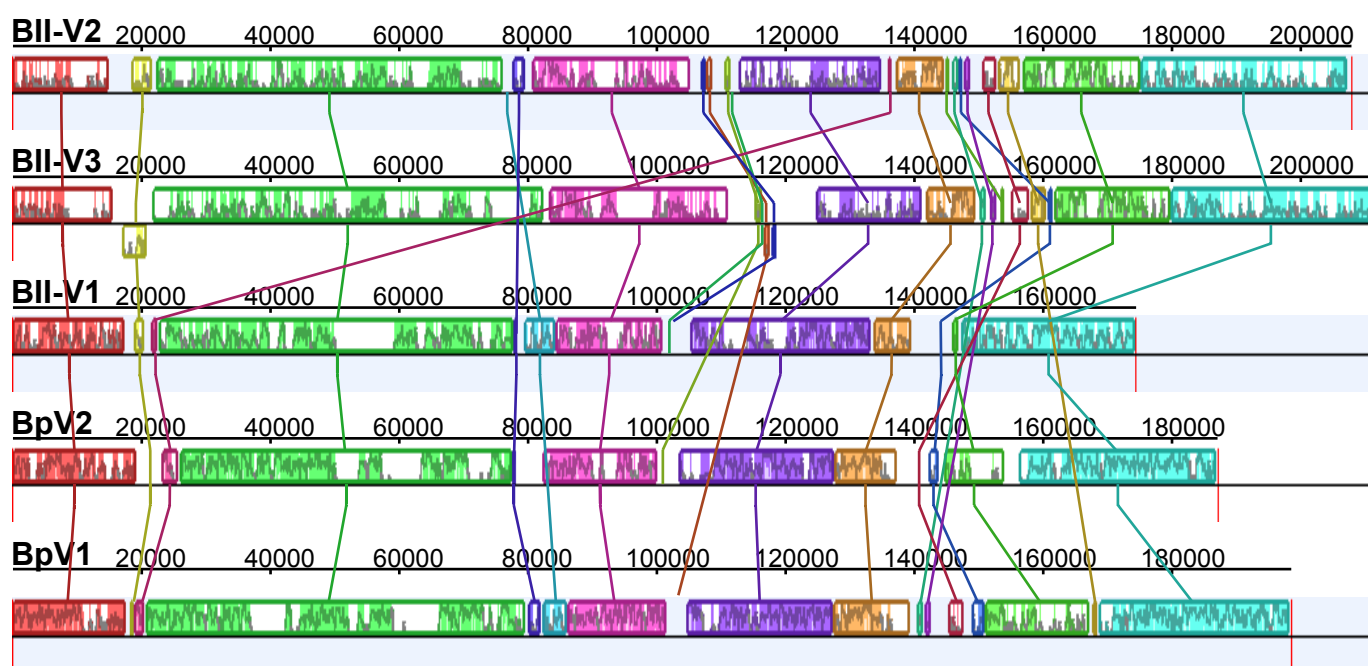

Figure S1.

Supplement: Supplementary file 2 — Figure S1 [file 41396_2021_989_MOESM2_ESM.pdf]

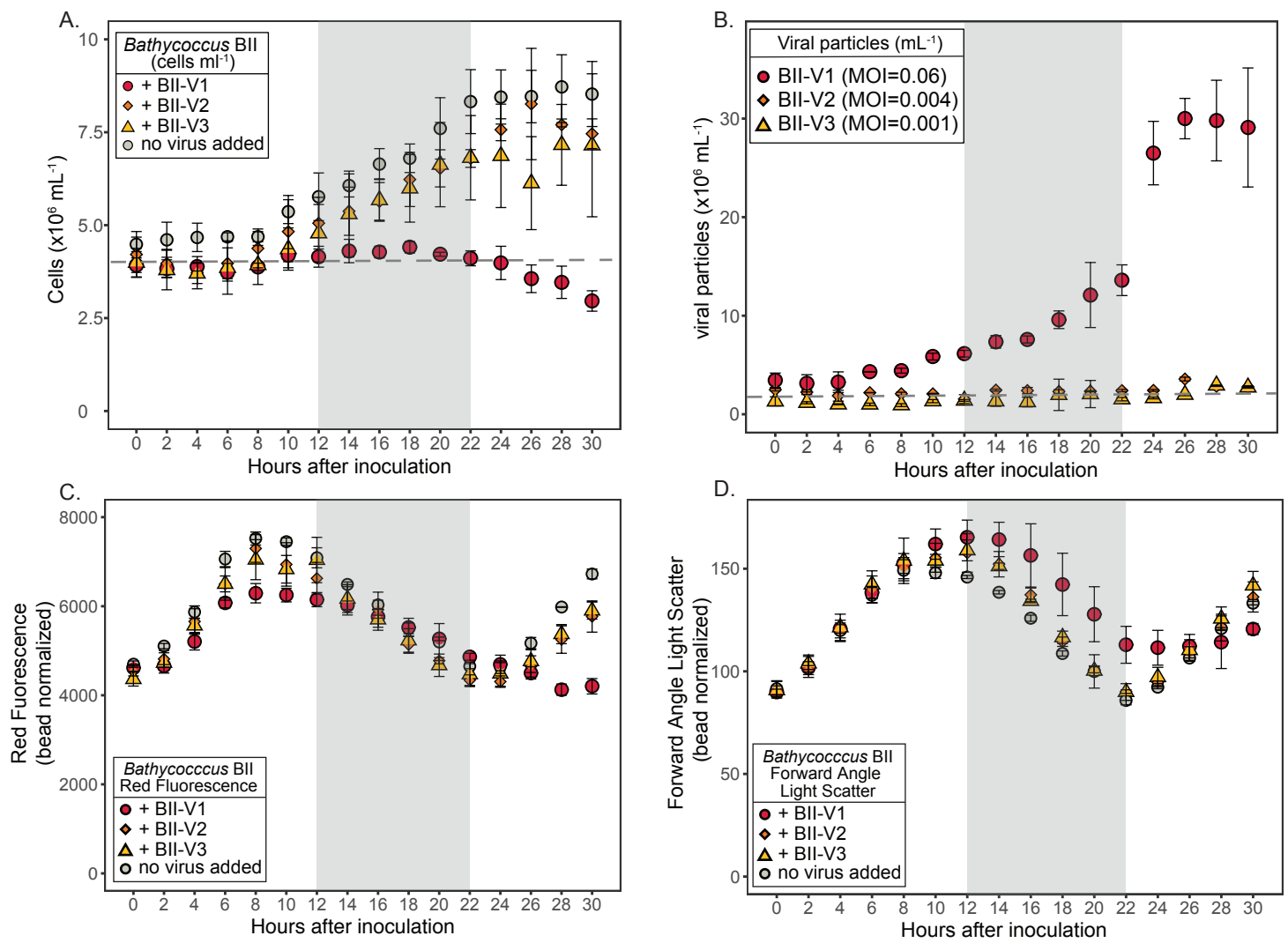

Figure S2.

Supplement: Supplementary file 3 — Figure S2 [file 41396_2021_989_MOESM3_ESM.pdf]

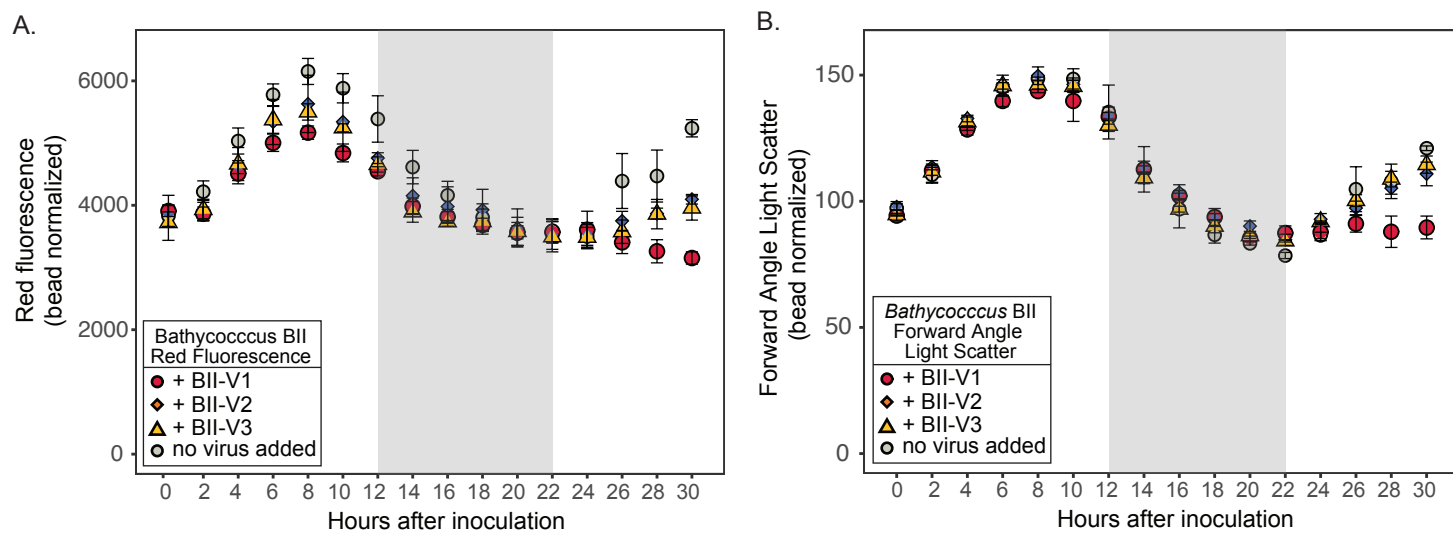

Figure S3.

Supplement: Supplementary file 4 — Figure S3 [file 41396_2021_989_MOESM4_ESM.pdf]

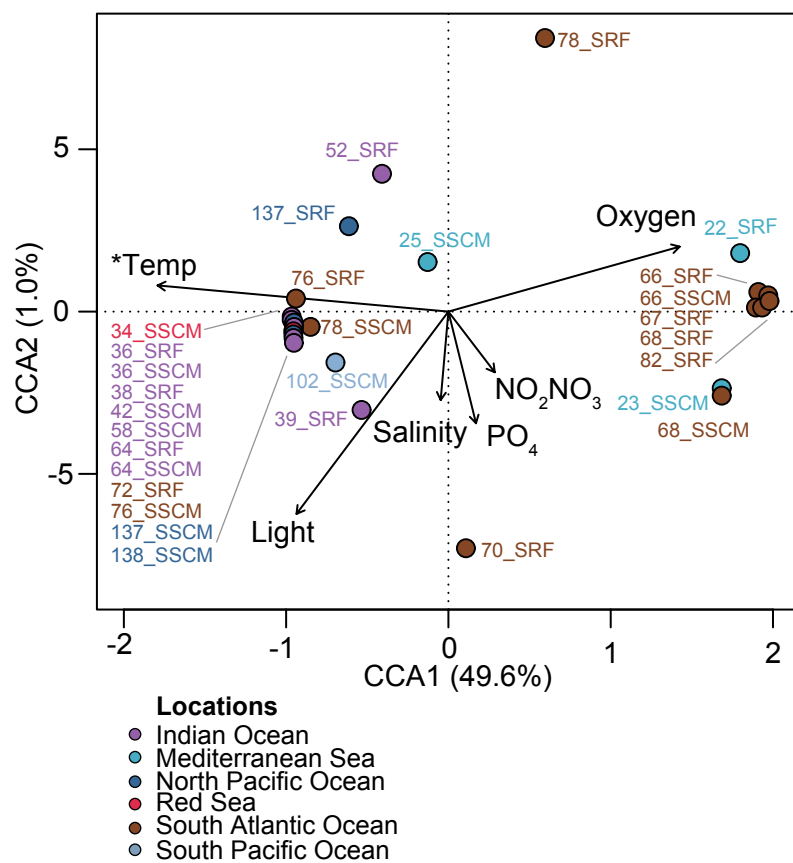

Figure S4.

Supplement: Supplementary file 5 — Figure S4 [file 41396_2021_989_MOESM5_ESM.pdf]

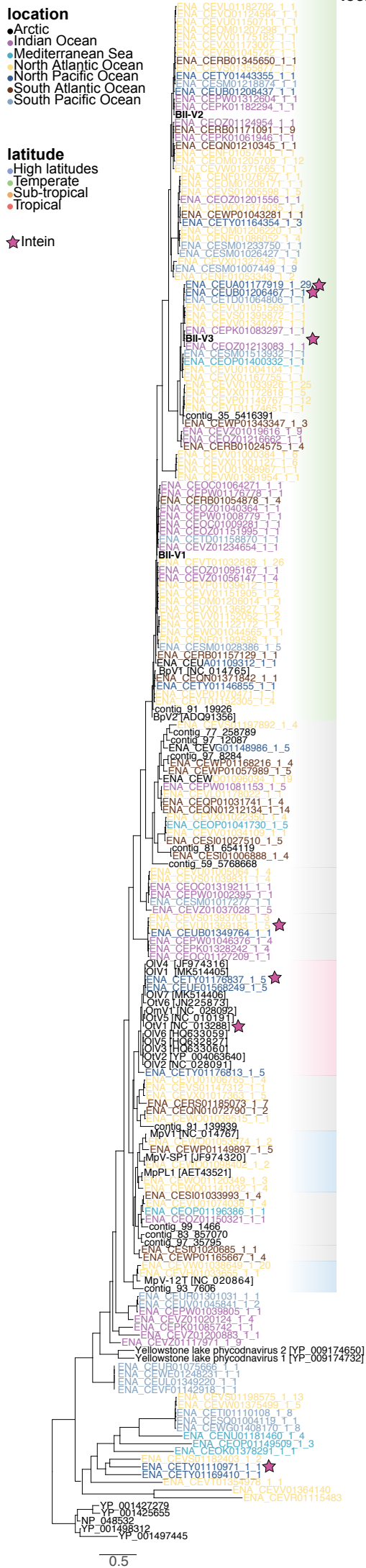

location latitude

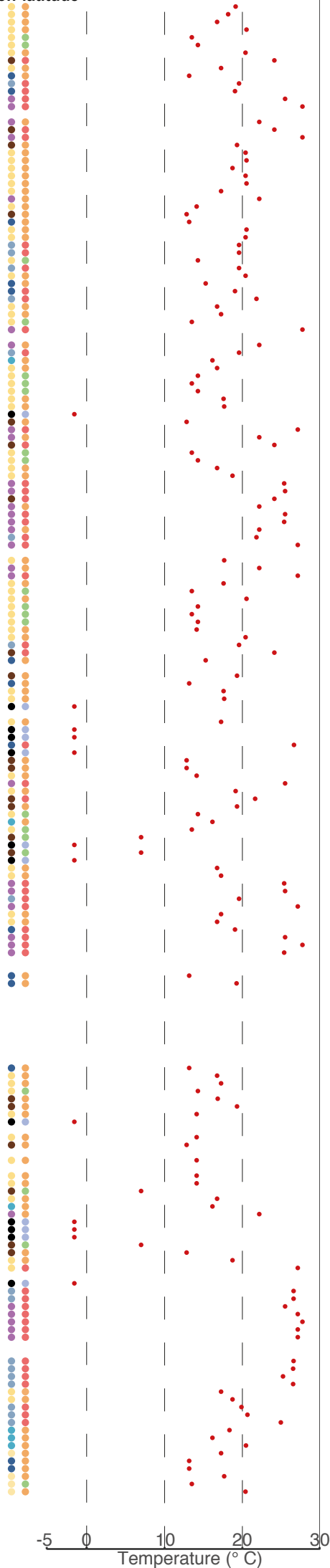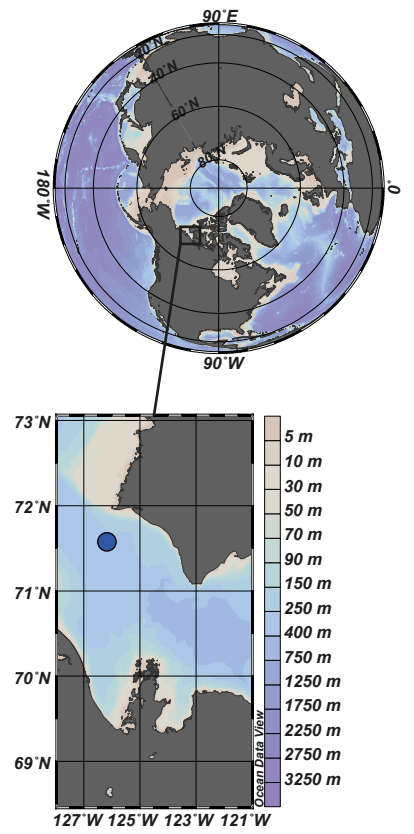

Figure S5.

Supplement: Supplementary file 6 — Figure S5 [file 41396_2021_989_MOESM6_ESM.pdf]
