# Supplementary material for: Viruses infecting a warm water picoeukaryote shed light on spatial co-occurrence dynamics of marine viruses and their hosts
Source: ISME J. 2021 May 11;15(11):3129–47. doi: 10.1038/s41396-021-00989-9 (PMC8528832; doi:10.1038/s41396-021-00989-9)
Supplement: Supplementary file 7 — Tables S1-S4-S5 [file 41396_2021_989_MOESM7_ESM.pdf]

Table S1 . List of viral genomes used for phylogenomics, orthology and functional categories (EggNOG) analyses in the present study.

Virus short name	Host species	Host Strain	Virus isolation reference	Access number
BpV2	Bathycoccus prasinos	RCC1105	Moreau et al. 2010	HM004430.1
BpV1	Bathycoccus prasinos	RCC1105	Moreau et al. 2010	HM004432.1
BII-V1	Bathycoccus calidus	RCC716	this study	MK522034-37
BII-V2	Bathycoccus calidus	RCC716	this study	MK522038
BII-V3	Bathycoccus calidus	RCC716	this study	MK522039
MpV-12T	Micromonas commoda	LAC38	Martinez Martinez 2015	SRS172696
MpV-PL1	Micromonas pusilla	CCMP1545 (i.e. UTEX 991, Plymouth 27)	Cotrell and Suttle 1991	HQ633072.1
MpV-SP1	Micromonas pusilla	CCMP1545 (i.e. UTEX 991, Plymouth 27)	Cotrell and Suttle 1991	JF974320.1
MpV1	Micromonas candidate species 2	RCC1109	Moreau et al. 2010	NC_014767.1
OtV6	Ostreococcus tauri	OTH 95	Monier et al. 2016	JN225873.1
OlV4	Ostreococcus lucimarinus	CCMP2972	Derelle et al. 2015	JF974316.1
OtV1	Ostreococcus tauri	OTH 95	Weynberg et al. 2009	FN386611.1
OtV5	Ostreococcus tauri	OTH 95	Derelle et al. 2008	EU304328.2
OmV1	Ostreococcus mediterraneus	RCC2590	Derelle et al. 2015	NC_028092.1
OlV7	Ostreococcus lucimarinus	CCMP2972	Derelle et al. 2015	MK514406
OlV1	Ostreococcus lucimarinus	CCMP2972	Derelle et al. 2015	MK514405
OlV2	Ostreococcus lucimarinus	CCMP2972	Derelle et al. 2015	NC_028091.1
OlV3	Ostreococcus lucimarinus	CCMP2972	Derelle et al. 2015	HQ633060.1
OtV2	Ostreococcus sp.	RCC393	Weynberg et al. 2011	FN600414.1
OlV5	Ostreococcus lucimarinus	CCMP2972	Derelle et al. 2015	NC_020852.1
OlV6	Ostreococcus lucimarinus	CCMP2972	Derelle et al. 2015	HQ633059.1
AtCV1	Chlorella sp.	SAG 3.83	Fitzgerald et al. 2007	NC_008724.1
PbCV1	Chlorella variabilis	NC64A	Van Etten et al. 1983	NC_000852.5
PbCVAR158	Chlorella variabilis	NC64A	Fitzgerald et al. 2007	DQ491003.1
PbCVFR483	Chlorella Pbi	ND	Fitzgerald et al. 2007	DQ890022.1
PbCVNY2A	Chlorella variabilis	NC64A	Fitzgerald et al. 2007	DQ491002.1

Table S4. Summary of the infection experiment parameters

Host	RCC716			RCC715		
Virus	BII-V1	BII-V2	BII-V3	BII-V1	BII-V2	BII-V3
Infectivity	3.5% (2.0%-6%)	4.9% (3.1%-7.9%)	0.5% (0.3%-0.8%)	1.8% (0.8%-2.9%)	0.06% (0.05%-0.08%)	0.03% (0.01%-0.04%)
Virus : host	4.6 ± 0.3	4.6 ± 0.6	1.2 ± 0.1	8.8 ± 0.4	6.0 ± 0.8	3.2 ± 0.5
MOI (95% CI)	0.10 (0.06-0.18)	0.10 (0.06-0.16)	0.01 (0.007-0.018)	0.06 (0.03-0.10)	0.004 (0.003-0.005)	0.001 (0.0003-0.0013)
*Latent period (Hr)	12	14	18	14	No significant diverge	No significant diverge
**Burst size	330 ± 267	32 ± 3	140 ± 23	136 ± 39	No significant diverge	No significant diverge

* Viral particle abundance started to show a significant diverge from starting abundances (T-test, $p<0.05$).

**Estimated from the increase in free viruses and the loss of host cells between the start of latent period and the time when the increase in viral abundance leveled off (n = 3). Units are progeny virions per cell.

TableS5. Viral-induced host mortality

	Host	
Virus	RCC716	RCC715
BII-V1	69% ± 13%	65% ± 6%
BII-V2	53% ± 7%	12% ± 12%
BII-V3	35% ± 15%	15% ± 28%
